# Subsequent pregnancy after stillbirth: a qualitative narrative analysis of Canadian families’ experiences

**DOI:** 10.1186/s12884-023-05533-5

**Published:** 2023-03-27

**Authors:** Sarah Gower, Justice Luddington, Deep Khosa, Abhinand Thaivalappil, Andrew Papadopoulos

**Affiliations:** Department of Population Medicine, University of Guelph, 50 Stone Rd E, Guelph, ON N1G 2W1 Canada

**Keywords:** Stillbirth, Subsequent pregnancy after stillbirth, Qualitative study, Canada, Narrative analysis

## Abstract

**Background:**

In Canada, nearly nine pregnancies end in stillbirth daily. Most of these families will go on to have subsequent pregnancies, but research into how best to care for these parents is lacking. This study explores the lived experiences and the most important aspects of person-centred care for Canadian families experiencing a pregnancy after a stillbirth.

**Methods:**

This qualitative descriptive design used secondary data collected from an online, international survey for bereaved parents who reported having experienced a pregnancy subsequent to a stillbirth. Only parents who identified as Canadian were included in this study. Three open text questions were asked about parents’ experiences in their subsequent pregnancy. An inductive thematic analysis approach was used with open coding and a constant comparative method.

**Results:**

Families’ responses fell into six main themes that identified what they would have preferred for high quality, excellent care. These included: (1) recognizing anxiety throughout the subsequent pregnancy, (2) wanting one’s voices and concerns to be heard and taken seriously, (3) needing additional and specific clinical care for reassurance, (4) desiring kindness and empathy from caregivers and others, (5) seeking support from others who had also experienced pregnancy after stillbirth; and (6) addressing mixed emotions including guilt, continuity of care and carer, positive thoughts versus more realistic ones, and poignant feelings of self-blame.

**Conclusions:**

Participants’ responses identified that pregnancy after stillbirth is an extremely stressful time requiring patient-oriented care and support, both physically and psychologically. Families were able to articulate specific areas that would have improved the experience of their subsequent pregnancy. Parents asked for high-quality clinical and psychosocial prenatal care that was specific to them having experienced a prior stillbirth. They also requested connections to others experiencing this similar scenario. Further research is needed to delineate what supports and resources would be needed to ensure this care would be available to all families experiencing pregnancy after stillbirth across Canada and their caregivers.

## Background

Stillbirth is a devastating outcome of pregnancy that affects thousands of families every year. Many parents start to think about another pregnancy very early on, even in the first hours and days of learning their current pregnancy is over [1]. Between 50 and 80% of families experiencing stillbirth will become pregnant again [2], and most of those will be within the first 12 to 18 months [3–5]. However, patients consistently report increased levels of anxiety and depression in their subsequent pregnancies [2, 6–8], as do their partners [1, 9, 10]. Patients report feeling increased vigilance towards the subsequent pregnancy, having flashbacks to their previous loss, feeling an increased sense of risk and danger, and a reactivation of the grief and helplessness they experienced when their previous baby died [3, 6]. Each subsequent pregnancy loss, including early miscarriage, increases these symptoms [2].

Meanwhile, family, friends and caregivers often assume the subsequent pregnancy and baby will heal all and make everything better again, but this is not usually the case [11, 12]. Similarly, symptoms of anxiety and depression are rarely resolved with the birth of a live baby [2]. Parents experience prolonged grief like that following the loss of a child of any age [7], and families are at a high risk of developing complicated grief and post-traumatic stress disorder [13, 14].

Patients who have experienced one stillbirth have up to a five-times greater chance of recurrent stillbirth [15]. Further, a previous stillbirth increases the risk of other serious complications in a subsequent pregnancy including low birthweight, preterm labour, and neonatal mortality [16]. These outcomes do vary widely depending on what the cause of the initial stillbirth was deemed to be [17]; however, at least 50% of stillbirths worldwide continue to have an unknown cause.

The Lancet’s “Stillbirths Series” in 2011 identified stillbirth as “one of the most shamefully neglected areas of public health” (pg. 1550) and, among other things, recommended increased investment in relevant research [18]. The global economic, psychological, physical and economic burden of stillbirth is a universal problem which is under-researched and under-resourced [18–20]. Various studies have looked at clinical [21] and psychosocial [4] aspects of pregnancy after stillbirth; however, there is a lack of strong evidence to guide care. In particular, there is a lack of patient-centred or patient-oriented research in terms of perspectives of the type of care families would value or have valued in a subsequent pregnancy.

In 2015, partly in response to the 2011 Lancet series, Heazell et al. undertook a process to determine current research priorities for stillbirth. Through an iterative process that began online and concluded with a one-day workshop, they brought together clinicians, bereaved parents, researchers and nonprofit groups to generate and eventually narrow down a list of high priority research questions. One such question was “How can staff support women and their partners in subsequent pregnancies, using a holistic approach to reduce anxiety, stress and any associated increased visits to healthcare settings?” [22]. The present study will contribute to answering this question by exploring the lived experiences of Canadian families during a pregnancy subsequent to stillbirth and the perceptions of the most important aspects of person-centred care during subsequent pregnancies among these families.

## Methods

### Design

This study was a qualitative descriptive design using secondary data accessed through ethics approvals and data-sharing agreements between the Mater Research Institute – The University of Queensland (MRI-UQ) in Brisbane, Australia. where the data was housed, and the University of Guelph in Ontario, Canada, where the data was analyzed. All data were anonymized by MRI-UQ prior to sharing and were therefore non-identifiable. Files were encrypted during transport and securely stored on an encrypted password protected computer during analysis.

### Data collection

As part of the 2016 Ending Preventable Stillbirths Series [23], MRI-UQ and the International Stillbirth Alliance together developed three multi-language web-based surveys. Each was geared towards a different population: bereaved parents, care providers, and general community members. Links to the online survey were disseminated online through International Stillbirth Alliance member organizations, charity organizations, and additional relevant professional societies between December 2014 and February 2015. Specific to Canada, these organizations included the International Confederation of Midwives, International Federation of Gynaecology and Obstetrics, Angel Whispers Baby Loss Support Program, Parent Care Society of Edmonton, Walk To Remember, and Still Life Canada. Due to the nature of this online recruitment, we were unable to determine the response rate and how many men and women viewed the study advertisements [23]. For further details on the survey methods, see Wojcieszek et al. (2018) [24].

The secondary data used in this study were from the survey distributed specifically to bereaved parents [5]. This study analyzes a section which was available only to respondents who reported a subsequent pregnancy after their stillborn baby. Three open-text questions were asked about perceptions of care in the subsequent pregnancy after experiencing a stillbirth: what was most important in that care, ways their care could have been improved, and an “anything else” question (Table 1). Categorical questions from this section have been previously analyzed and published [5].

**Table 1:** Open-text questions asked to respondents who had experienced a pregnancy after stillbirth

Item	Question
1	Thinking about the care you received in the pregnancy after your baby was stillborn, what were the most important things to you?
2	Are there ways your care could have improved in the pregnancy after your baby was stillborn?
3	Is there anything else you would like to tell us about the pregnancy after your baby was stillborn?

### Data selection

In total, there were 4182 independent respondents from 32 different countries. Initial descriptive statistics were generated from this data and sorted by country. This showed that while many countries had only a few respondents, others had a high number of respondents overall, but a low response rate to the open-text qualitative questions (Table 2). Canada had the twelfth highest raw number of respondents overall, but many answers from all countries included “no” or “nothing”. In considering the percent of “non-no” responses, Canada was among the top five. (Table 2). Further advantages were that an all-Canadian response set would allow the analysis of the Canadian healthcare system response to childbirth. As well, 98% of the Canadian answers were in English (2% in Spanish), minimizing translation requirements. In consultation with the research team, it was decided to use only the Canadian data for the purposes of this study.

**Table 2:** Response rates to open-text questions from the five highest “non-no” responding English-speaking countries

Rank	Country	Respondents (n)^a^	Replies to Q1-Q3 (n)^a^	Response rate (%)^a^
1	Sweden	133	89	66.9
			16	12.0
			36	27.0
2	Republic of Ireland	130	75	57.7
			25	19.2
			41	31.5
3	United States	402	182	45.3
			134	33.3
			149	4.7
4	Canada	62	24	38.71
			18	29.03
			17	27.42
5	Australia	444	33	7.4
			18	4.1
			20	4.5

^a^ Excludes responses which simply stated “no” or “nothing”

### Data analysis

The Canadian dataset was imported into NVivo 12 (QSR International, Doncaster, Australia) for organization and analysis. Transcripts were read multiple times by two independent reviewers (SG, JL) and subsequently analyzed under a social constructivist framework. Social constructivism accepts that there is both a subjective and objective reality; an objective world exists but is subjectively constructed through social interaction and experience [25]. Specifically, an inductive thematic analysis was used with an open coding framework [26, 27] and a constant comparative method [28]. Code words or phrases were applied to sections of text to represent concepts that participants described. This was an iterative process with the respondents’ text read and reread multiple times with comparison against existing code phrases and themes. Both reviewers performed this independently. Responses to questions 1, 2 and 3 were kept separate at this point in the process.

One reviewer (SG) then regrouped similar codewords and phrases together and renamed them into themes and subthemes. Themes generated from this step were then reviewed and agreed on through discussion by the second reviewer (JL). The final step involved a comprehensive review of all themes across the three sections, and one reviewer (SG) generated five overarching themes as well as some additional specific points that were felt to be worth highlighting. To enhance credibility of data analysis, all team members were invited to review the findings and provide suggestions for amendment. A third researcher (DK) suggested additional points be combined into a sixth theme, and this was agreed to by both initial reviewers. Results were then organized using the final six coded themes, with representative quotations used for illustration. This methodology allowed the patients’ thoughts, words, and experiences to remain central to the findings, and ensured the results would be acutely relevant [29] for the aims of this study.

## Results

The initial complete data set included responses to the entire stillbirth questionnaire from 32 high-income countries, resulting in 4182 individual respondents in total [23]. The original “pregnancy after stillbirth” dataset included responses from 2716 individuals (2507 female, 204 male, 5 not stated). There were 62 Canadian respondents with a response rate of 40.3%, 38.7% and 29.5% to the three open-text questions, respectively (Table 2). Demographics of the Canadian respondents are included in Table 3.

**Table 3:** Demographic and obstetrical characteristics of Canadian respondents to “subsequent pregnancy” open-text questions (n = 62)

Characteristic	N (%)
Age range	
< 20	0 (0)
20–29	8 (12.9)
30–39	34 (54.8)
> 40	20 (32.2)
Not stated	0 (0)
Gender	
Male	6 (9.6)
Female	56 (90.3)
Not stated	0 (0)
Highest educational level	
No formal qualifications	0 (0)
Secondary/High school	5 (8.1)
Undergraduate/college degree	41 (66.1)
Post-graduate degree	14 (22.6)
Trade, apprenticeship, other	2 (3.2)
Not stated	0 (0)
Employment	
Not employed	1 (1.6)
Employed part-time	10 (16.1)
Employed full-time	37 (59.7)
Homemaker, student, retired, other	14 (22.6)
Not stated	0 (0)
Interval between index stillbirth and survey completion	
0–11 months ago	15 (24.2)
1–2 years ago	24 (38.7)
3–5 years ago	11 (17.7)
5–9 years ago	12 (19.4)
Not stated	0 (0)
Gestation at index stillbirth (weeks)	
< 24	8 (12.9)
25–29	10 (16.1)
30–34	6 (9.7)
35–37	10 (16.1)
38–40	21 (33.9)
> 40	7 (11.3)
Not stated	0 (0)
Deaths of other children from any cause before stillborn baby	
Yes	16 (25.8)
No	33 (53.2)
Not applicable	13 (21.0)

### Themes

The following sections describe six major themes around the care parents would have liked to have received during a subsequent pregnancy after stillbirth (Table 4). Quotations are labelled by respondent number as assigned to them within the original full survey. Analysis was conducted on responses from all three open-text questions and subsequently, the themes encompassed responses from all three questions.

**Table 4:** Themes and representative quotes around what respondents would have preferred for care in subsequent pregnancy

No.	Theme	Representative quote
1	Recognizing anxiety throughout the subsequent pregnancy	*“An anxiety-provoking experience”* (7943)
2	Wanting one’s voices and concerns to be heard and taken seriously	*“I’m taken seriously if I think something is wrong.”* (8809)
3	Needing additional and specific clinical care for reassurance	*“Extra ultrasounds, for peace of mind”* (8656)
4	Desiring kindness and empathy from caregivers and others	*“Kindness and respect from my care provider”* (7239)
5	Seeking support from others who had also experienced pregnancy after stillbirth	*“I am also wanting some counselling specific to pregnancy after loss.”* (8662)
6	Addressing mixed emotions including guilt, continuity of care and carer, positive thoughts versus more realistic ones, and poignant feelings of self-blame	*“Very overwhelming, emotional and beautiful. Hard to grasp”* (21,279)

### Theme 1: recognizing anxiety throughout the subsequent pregnancy

An overwhelming anxiety that the present pregnancy would also end in stillbirth permeated virtually every response to the open-text questions. This was expressed in terms such as *“anxiety”*, *“stressful”* (22,358), *“overwhelming”*, *“extremely distressing”* (8741) and *“horribly terrifying”* (8664). Individuals often mentioned a range of negative emotions:

*“An Anxiety-Provoking Experience.”* (7943).

*“It was absolutely the most nerve-racking nine months of my life, constantly worrying that this baby may die too.”* (11,251).

*“It was the scariest time of my life.”* (8127).

Respondents expressed they needed *“appreciation for how stressful it was”* (22,358) and were constantly trying to find *“peace of mind”*. One respondent, when asked what was most important in her care, simply wrote *“The baby survived”* (20,488), summing up many of the other respondents’ greatest fears that their baby would not survive.

### Theme 2: wanting one’s voices and concerns to be heard and taken seriously

A second theme was the importance of not having their concerns during this pregnancy brushed off, and *“not dismissing them as silly”* (8664).

*“I had an OB that listened to my concerns and didn’t judge she answered my questions and was absolutely amazing.”* (11,251).

*“I’m taken seriously if I think something is wrong.”* (8809).

Related to this, patients were not satisfied to be told everything would be fine, as if they were in their first pregnancy. They saw themselves as distinct from parents who had not previously experienced this devastating outcome and did not want their previous experience *“minimized”* (7239). They had lost their naiveté about pregnancy, and it was clear they did not want to be dismissed around their very real fears of their current pregnancy ending with a dead baby:

*“I didn’t want anyone to sugarcoat anything. I didn’t want to be talked down to. I wanted it recognized that the pregnancy was not a sure thing.”* (8099).

This same respondent also commented:

*“I wish that that first pregnancy after stillbirth hadn’t been treated as if it were ‘of course’ going to result in a live baby; there is no guarantee of that and I knew it and I didn’t appreciate being treated as if I was being negative for thinking so.”* (8099).

The importance of being taken seriously was also directed at family and friends, not just caregivers:

*“It was very stressful. [My first baby] died during labour - so having good scans, a strong heartbeat - none of that was reassuring. So what? I had that before and my baby died. Not too many people understand that. I also felt that so many people think infant loss doesn’t happen in modern times was crazy-making. I had to argue with people that healthy babies do die and would spout statistics. Why do we hear so much about SIDS and so little about stillbirth?”* (7952)

### Theme 3: needing additional and specific clinical care for reassurance

Many respondents discussed changes in the frequency of their medical care in their subsequent pregnancy and saw this as positive. They appreciated *“more frequent appointments and monitoring”* (21,279), *“seeing care providers regularly, and frequent ultrasound reviewed by a perinatologist”* (7943), and in general *“extra care, which was helpful”* (8145). Even simple moments that brought reassurance, like hearing their baby’s heartbeat, seeing movements on ultrasound, and hearing that growth was normal (7239) were remembered. One individual commented:

*“Extra ultrasounds, for peace of mind.”* (8656).

On the other hand, sometimes extra testing led to more anxiety:

*“I was sent for a late scan to give me ‘peace of mind’ at about 36 weeks but the ultrasound tech panicked that my baby had a chubby neck and I was sent to a perinatologist at [BC Women’s Hospital] for a consult and another scan because they were worried there was something wrong. There wasn’t - so what should have given me peace of mind did not.”* (7952)

Delays or unavailable tests also created stress:

*“The option for a 10-week nuchal fold ultrasound covered by [insurance] would have gone a long way to ease my anxiety, rather than waiting for 20 weeks for a fetal echocardiogram.”* (8656)

Some felt that care in their subsequent pregnancy was not ideal. This quote came from a parent who had experienced two stillbirths:

*“…[this] baby was still stillborn. It was after this pregnancy they found out I have a blood clotting disorder. My babies should not have died. This was preventable if they had done the proper testing after my premature birth and especially after my first stillbirth.”* (7239)

One respondent noted that different care might have been useful in her index stillbirth pregnancy. She also mentioned buying a home fetal doppler monitor for more reassurance:

*“It was bittersweet that I had to lose one baby to get that kind of care i.e. more frequent scans.”* (11230)

### Theme 4: desiring kindness and empathy from caregivers and others

The fourth theme of care during subsequent pregnancy was a desire to simply be listened to:

*“Kindness and respect from my care provider”* (7239).

*“I had an OB that listened to my concerns and didn’t judge.”* (11,251).

*“My midwives acted more as counselors and listened to my concerns.”* (8127).

Others commented that they had these experiences and were pleasantly surprised by them:

*“…an obgyn who let me tell her my fears and walked me through it step by step with a hug.”* (8609)

*“The kindness and care given by my OB was above and beyond what I expected.”* (9827)

Phrases such as *“additional emotional care”* (9092) and *“help emotionally”* (8741) were used to express patients’ desires to be treated with empathy:

*“My doctor cared. She was on call when [name] was born and had a personal interest in my next 2 pregnancies. She took the afternoon off when I went in for an early c-section so that she could be there. She kept me sane. I was told to come in any time for anything. I knew that she would stand up for me if anyone else gave me grief.”* (7333)

On the other hand, if caregivers were remembered as having acted unkindly, this was perceived as a negative experience:

*“I don’t know if the outcome would have been different had [doctor] listened to me…He acted like I was being a big suck and said I should tie my tubes.”* (7952)

*“My OBGYN was not listening to my concerns.”* (8127)

These negative memories reinforce that simple empathetic listening was both remembered and highly valued:

### Theme 5: seeking support from others who had also experienced pregnancy after stillbirth

This theme centred around specific types of counselling and peer support for parents experiencing pregnancy after stillbirth. It was clear that, similar to parents reporting a need for different clinical care, many would also have appreciated a more tailored emotionally supportive care:

*“Specialized bereavement care for parents who had already experienced a stillbirth.”* (7239)

*“I am also wanting some counselling specific to pregnancy after loss.”* (8662)

*“It helped to connect with other moms who had a similar experience and successfully gave birth after they had a stillborn child.”* (7943)

One respondent mentioned a type of therapy specific to trauma survivors, Eye Movement Desensitization Reprocessing and wished she had discovered it earlier in her pregnancy (7943). It was very clear that these people did not find it helpful to attend groups including families who had not experienced stillbirth:

*“I needed counselling with someone specializing in pregnancy after loss, and a dedicated support group. I did not feel comfortable going to a regular support group while pregnant.”* (8127)

### Theme 6: Addressing mixed emotions including guilt, continuity of care and carer, positive thoughts versus more realistic ones, and poignant feelings of self-blame

A sixth and final theme encapsulated mixed emotions and responses expressed in different and even contradictory ways. Several respondents noted they felt like hiding their pregnancy to avoid having to act happy and hear false reassurances:

*“I didn’t announce the pregnancy to people or family out of town until [I was six months pregnant]…I would have kept it secret to the end if I could have. I found the platitudes and promises that with prayer she would be fine really upsetting.”* (7952)

*“It was extremely stressful feeling like I had to pretend to be happy for everyone else. When what I really wanted was to hide until a healthy baby was born.”* (8656)

Other respondents looked for *“hope and positivity”* (9880) from family, friends and caregivers while simultaneously wanting their concerns taken seriously – they wanted realistic but positive support. Some even mentioned the complexity of the situation by expressing multiple feelings:

*“Very overwhelming, emotional and beautiful. Hard to grasp”* (21279)

Regarding continuity of care and carer, some felt strongly they wanted the same type of care or care provider as in their pregnancy that had ended in stillbirth (8145, 7952), others equally wanted a different obstetrician (8099) or a different type of anesthetic (22,358). Two respondents mentioned that caregivers were not always aware of their prior stillbirths. They found this quite challenging as they had to periodically re-explain their previous traumatic experience (7239, 8145).

Finally, there were some particularly poignant responses which stood out to our research team. One respondent stated that her care would have been improved by *“speaking up when I had thoughts”* (8609), reflecting her own guilt and uncertainly around her pregnancy. Another answered that one of the most important parts of her care was simply *“acknowledgement of my baby who was stillborn”* (7239). A third highlighted the following:

*“…the feeling that the mother is a ‘failure’ and ‘is not meant to bear healthy babies’. Physical care aside, what goes on in the mother’s mind was a true challenge to break through.”* (9092)

## Discussion

Our study explored Canadian parents’ experiences in a subsequent pregnancy after stillbirth. The findings resulted in six main themes that highlighted what they would have preferred for high quality, excellent care. The main areas were around recognizing anxiety throughout the subsequent pregnancy; wanting one’s voices and concerns to be heard and taken seriously; needing additional and specific clinical care for reassurance; desiring kindness and empathy from caregivers and others; seeking support from other families who experienced pregnancy after stillbirth; and addressing mixed emotions.

Previous analysis of the categorical data from the source dataset identified that while parents frequently had additional care in the form of prenatal visits and ultrasounds, they did not feel well supported psychosocially during their subsequent pregnancy [5]. This highlights that clinical care encompasses more than clinical services, and that maternity services and individual clinicians need to consider how they could improve their care. The results of the current study begin to identify why these parents did not feel supported and what could have improved their care.

Perinatal bereavement care and supports specific to a subsequent pregnancy after stillbirth is essential for this group. Families dealing with infant loss can experience intense negative emotions with the bereavement period extending long-term inducing major life changes to mental health, relationships, employment, and core beliefs [6–8]. During a highly anxious pregnancy, these patients require extra clinical care, non-judgemental listening, and to be taken seriously about any concerns. These findings are consistent with the literature on psychosocial experiences of pregnancy after stillbirth [2–4, 6–8]. This should not be enormously different from patient-centred care and communication, which is a central and important tenet of current medical education [30, 31], but clearly there are barriers preventing some clinicians from providing this to these patients. Some providers may not have gained the communication skills to enable a patient to feel comfortable disclosing their true fears. Some providers may fear bringing up certain topics, worried that they may remind the patient of something they would rather not discuss. Health care professionals can struggle with uncertainty, especially when the stakes feel high in a subsequent pregnancy [32]. Our data shows that patients would prefer professionals to communicate openly, with empathy and to discuss patients’ previous experiences and current fears. Ironically, these discussions may in fact be an effective therapeutic intervention, rather than pretending all is well or downplaying patients’ concerns. Until recently [4], clinical guidelines surrounding pregnancy after stillbirth were primarily medical in nature and only briefly mentioned the need for emotional care on the part of the caregiver [33–35]. This must be strongly emphasized in clinical guidelines of care after stillbirth [11], with more recent Canadian guidelines emphasizing psychosocial and peer support for women and families who experience stillbirth [36]. These guidelines also provide some evidence on considerations for providers to communicate with patients who have had prior adverse pregnancy outcomes and who have uncertain prognoses [36], and these components can be integrated within health care training to support providers to deliver high-quality care. Further research aimed at best practices and barriers in teaching authentic emotional communication to practitioners both in terms of its importance and the skills themselves are essential and can advance care provided to families [36–38].

Connecting with others who have been through a pregnancy after a stillbirth and receiving support and advice from them was also deemed essential. Specifically, “regular” prenatal groups were not found to be useful. Families who have experienced a stillbirth deal with a level of anxiety and fear during their current pregnancy that other pregnant families may not be able to appreciate. Further, they may hesitate to bring up their experiences or true worries for fear of alarming other parents. There may be therapeutic outcomes to cohort-specific support groups, whether peer-led or professional-led; both Clifford and Lidell reported a higher live birth rate in patients who had experiencing recurrent miscarriages with targeted emotional peer support [39, 40]. Additionally, a previously published survey from Canada found that families who accessed peer supports found it helpful to share their experiences and hear stories from others who experienced infant loss [41]. Online support groups have also been studied [42–44] and found equally or more beneficial than in-person support. Haik [44] investigated an online group for a rare medical condition and found that “a highly moderated group with interactions focused on treatment information, emotional support and expression, and community building” was helpful, because it allowed “patients with rare disease to overcome geographical and temporal boundaries to connect and share experiences and resources with others in the same cohort” (p.298). A qualitative study based out of Canada also revealed a combination of support systems for families (e.g., medical support, support groups, counseling) after stillbirth can help dealing with the process in the short- and long-term [45], shape the pregnancy experience [41], and these supports may be extended to subsequent pregnancy as well. Further exploration into the improved provision this cohort-specific support to all families experiencing pregnancy after stillbirth is recommended.

An important question these results generate is whether this *specific* care also needs to be *specialized* care requiring sub-specialist involvement, or whether this care can be delivered at a primary care, community level through an interdisciplinary team. Although the findings from our study only hint at it, the evidence other literature supports the need for both [45, 48], where some patients may prefer new caregivers and a more specialized feel to their pregnancy while others may find it more reassuring to stay in their home region with caregivers who know their history well and can offer support in familiar surroundings without lengthy travel. Family physicians are trained to expertly manage uncertainty of clinical outcomes [46, 47], advise on known risk factors [48], and provide patient-centred communication across a patient’s entire lifespan [49, 50]. They follow these families for decades before and afterwards and see the patient not only through her index and subsequent pregnancies, but watch those children grow up, as well as care for extended family members each grappling with their own experiences of life after a stillbirth. There is a real, evidence-based value in maternity care close to home [51] and while some pregnancies might require highly specialized obstetrical or psychological care, many of the elements the families in this study discussed could be made available in community, rural and remote settings with proper support for clinicians.

It is worth noting that countries follow different definitions for what constitutes as stillbirth, and this impacts surveillance as well downstream services. Most provinces in Canada, except Quebec, have criteria for stillbirth which include gestational age of ≥ 20 weeks and ≥ 500 g [52]. There have been calls to update this definition in Canada and other criteria (e.g., to not classify induced abortions due to congenital anomalies as stillbirths) to follow guidelines from other countries [53]. For the purposes of this study which aims to explore experiences, the nature of stillbirth and the resulting services experienced by Canadian families can and likely do vary across the country and thus, we recommend patient care be mindful of this as well.

### Limitations and strengths

There are limitations to our study. First, we conducted a qualitative study embedded within a longer, primarily quantitative survey, rather than designed independently as purely qualitative. Concerns have been raised in the literature as to whether analyzing free-text responses can be considered qualitative research results [53, 54]. The online format also meant that we were unable to engage in a dialogue with participants to clarify their answers and find true data saturation as is more achievable with focus groups or personal interviews. The survey was designed to keep participants unidentified, such that there was no opportunity to contact respondents for follow-up questions. This limited our ability to conduct member checking to enhance the trustworthiness of our findings. Additionally, the survey component did not collect some demographic data such as province of residence, ethnicity, and income. We acknowledge that stillbirth in Canada is a nuanced topic with varying definitions [52], services which vary across regions, and familial experiences that may not be equitable across all groups. This limits us in commenting on the generalizability of our findings. Most respondents in this survey were employed and well-educated, and these experiences may differ from other groups. We advise future qualitative inquiries in this topic collect more demographic characteristics to allow possible identification of region- and group-specific challenges from families. Lastly, recruitment was achieved by using online approaches which involved distributing surveys through support groups and charity organizations. Therefore, it is possible the participants in our study may not be representative of the broader Canadian population of parents who have had stillbirth.

However, there were several strengths of this method of qualitative analysis for the purposes of our research objectives. Stillbirth is an intensely emotional and private event, and patients may have felt more comfortable answering questions at their own pace, in the privacy of their homes, rather than speaking in front of researchers and other participants in more clinical or office-based settings. The questions were phrased deliberately to try and elicit a variety of answers and were not just the “any other comments” that are sometimes tacked on to quantitative research surveys [55, 56]. And most importantly, we were able to apply a robust qualitative analysis to the data as the central focus of our study. This study stands as an independent adjunct to the previously published quantitative analysis [24], gives us rich insight into the lived experiences of families, and, importantly, allows us to develop practical key messages, and relevant questions for further research.

## Conclusions

Pregnancy after stillbirth is an extremely stressful time that deserves and requires specific patient-oriented care and support, both physically and psychologically. In this study, families were able to articulate specific areas that would have improved the experience of their subsequent pregnancy. Parents asked for high-quality clinical and psychosocial prenatal care that was specific to them having experienced a prior stillbirth. They also requested connections to others experiencing this similar scenario. Further research is needed to delineate what supports and resources would be needed to ensure this care would be available to all families experiencing pregnancy after stillbirth across Canada.

## Data Availability

The datasets used and analyzed during the current study were through secondary use with permission received from the Mater Research Institute – The University of Queensland (MRI-UQ). The authors do not have permission to share this dataset. The original dataset may only be available upon reasonable request with the MRI-UQ by contacting Dr. Vicki Flenady at vicki.flenady@mater.uq.edu.au.
